# Epithelial–Mesenchymal Transition Suppresses AMPK and Sensitizes Cancer Cells to Pyroptosis under Energy Stress

**DOI:** 10.3390/cells11142208

**Published:** 2022-07-15

**Authors:** Mingwei Liang, Jennifer W. Li, Huacheng Luo, Sarah Lulu, Ozlem Calbay, Anitha Shenoy, Ming Tan, Brian K. Law, Shuang Huang, Tsan Sam Xiao, Hao Chen, Lizi Wu, Jia Chang, Jianrong Lu

**Affiliations:** 1 Department of Biochemistry and Molecular Biology, College of Medicine, University of Florida, Gainesville, FL 32610, USA; liang_mingwei@grmh-gdl.cn (M.L.); jennifer_w_li@brown.edu (J.W.L.); hluo1@pennstatehealth.psu.edu (H.L.); sarahlulu@usf.edu (S.L.); ashenoy@chsu.edu (A.S.); 2 Department of Anatomy and Cell Biology, College of Medicine, University of Florida, Gainesville, FL 32610, USA; ozlem@ufl.edu (O.C.); shuanghuang@ufl.edu (S.H.); 3 Graduate Institute of Biomedical Sciences and Research Center for Cancer Biology, China Medical University, Taichung 406040, Taiwan; mingtan@cmu.edu.tw; 4 Department of Pharmacology and Therapeutics, College of Medicine, University of Florida, Gainesville, FL 32610, USA; bklaw@ufl.edu; 5 Department of Pathology, Case Western Reserve University School of Medicine, Cleveland, OH 44106, USA; tsx@case.edu; 6 Department of General Surgery, Second Hospital of Lanzhou University, Lanzhou 730030, China; ery_chenh@lzu.edu.cn; 7 Department of Molecular Genetics and Microbiology, College of Medicine, University of Florida, Gainesville, FL 32610, USA; lzwu@ufl.edu; 8 Department of Periodontology, College of Dentistry, University of Florida, Gainesville, FL 32610, USA; jchang@dental.ufl.edu

**Keywords:** cancer, EMT, AMPK, energy stress, metformin, pyroptosis

## Abstract

Epithelial–mesenchymal transition (EMT) is implicated in tumor metastasis and therapeutic resistance. It remains a challenge to target cancer cells that have undergone EMT. The Snail family of key EMT-inducing transcription factors directly binds to and transcriptionally represses not only epithelial genes but also a myriad of additional genomic targets that may carry out significant biological functions. Therefore, we reasoned that EMT inherently causes various concomitant phenotypes, some of which may create targetable vulnerabilities for cancer treatment. In the present study, we found that Snail transcription factors bind to the promoters of multiple genes encoding subunits of the AMP-activated protein kinase (AMPK) complex, and expression of AMPK genes was markedly downregulated by EMT. Accordingly, high AMPK expression in tumors correlated with epithelial cell markers and low AMPK expression in tumors was strongly associated with adverse prognosis. AMPK is the principal sensor of cellular energy status. In response to energy stress, AMPK is activated and critically reprograms cellular metabolism to restore energy homeostasis and maintain cell survival. We showed that activation of AMPK by energy stress was severely impaired by EMT. Consequently, EMT cancer cells became hypersensitive to a variety of energy stress conditions and primarily underwent pyroptosis, a regulated form of necrotic cell death. Collectively, the study suggests that EMT impedes the activation of AMPK signaling induced by energy stress and sensitizes cancer cells to pyroptotic cell death under energy stress conditions. Therefore, while EMT promotes malignant progression, it concurrently induces collateral vulnerabilities that may be therapeutically exploited.

## 1. Introduction

Epithelial–mesenchymal transition (EMT) is a prototype of epithelial plasticity through which cells shed their epithelial features and acquire mesenchymal characteristics [1]. EMT is orchestrated by EMT-inducing transcription factors (EMT-TFs), mainly of the Snail, Zeb, and Twist families [1]. EMT enhances cell motility, invasiveness, stemness, and resistance to apoptosis. Aberrant induction of EMT in cancer has commonly been proposed to facilitate carcinoma metastasis and therapeutic resistance [2]. EMT-TFs are indispensable for metastasis in multiple genetically engineered mouse tumor models [3,4,5,6], and cancer cells undergoing partial EMT critically contribute to metastasis formation [7,8,9]. Studies in mouse tumor models validated that EMT promotes chemoresistance [10,11] and, in principle, resistance to molecularly targeted therapies against oncogenic drivers [12,13]. Clinical evidence supports that EMT confers resistance to front-line cancer treatments. In human breast cancer patients receiving endocrine therapy or chemotherapy, resistant cancer cells exhibited an EMT gene expression signature [14,15]. In human lung cancers treated with EGFR- or ALK-targeted therapies, EMT occurred in a subset of resistant cancers independent of genetic resistance mechanisms [16,17,18,19,20,21]. Due to its prominent role in cancer, EMT has emerged as a target of prime interest for therapy [22]. However, it is difficult to find cytotoxic agents that selectively kill cancer cells that have undergone EMT [23], in part because such cells have acquired increased resistance to apoptosis. No therapy specifically against EMT cancer cells has been established yet. It remains a challenging task to uncover specific vulnerabilities in EMT cancer cells for targeting.

EMT-TFs directly bind to and transcriptionally regulate a myriad of downstream target genes, which include not only classic epithelial and/or mesenchymal cell markers but also a large number of other genomic targets that may carry out diverse biological functions [24]. Accordingly, EMT involves global gene expression changes. In addition to down- and upregulation of typical epithelial and mesenchymal genes, respectively, expression of numerous additional genes is also concomitantly altered during EMT. Therefore, while EMT is characterized by the phenotypic change of epithelial cells to mesenchymal cells, it is also inherently accompanied by many other molecular processes as byproducts. Some of the EMT-associated changes may cause specific cellular deficiencies and make cells vulnerable to certain settings. Identification of such EMT-induced collateral vulnerabilities may enable selective targeting of EMT cancer cells.

A prerequisite for living cells is their ability to metabolically respond to low-nutrition conditions to maintain adequate energy supplies and sustain essential cellular functions [25]. Cells are equipped to sense energy stress. AMP-activated protein kinase (AMPK) is the principal sensor of cellular energy status across all eukaryotic species [26,27]. Mammalian AMPK is a heterotrimeric serine/threonine protein kinase complex consisting of one catalytic α-subunit (α1 or α2), one scaffolding β-subunit (β1 or β2), and one regulatory γ-subunit (γ1, γ2, or γ3). AMPK is activated by low energy status, signaled by rising intracellular AMP:ATP and ADP:ATP ratios. Activated AMPK phosphorylates a repertoire of downstream effectors to switch on ATP-generating catabolic processes (e.g., fatty acid oxidation, glycolysis, autophagy) to replenish ATP, while switching off ATP-consuming anabolic pathways (e.g., protein and lipid biosynthesis) [26,27]. AMPK-mediated metabolic reprogramming thus restores energy homeostasis and vitally maintains cell survival under energy stress conditions. Cells deficient in AMPK signaling fail to properly respond to energy stress and die from physiological metabolic stress or pharmacological compounds that target bioenergetic pathways [28,29,30].

There exist multiple programmed cell death pathways, including apoptosis, pyroptosis, ferroptosis, and necroptosis [31]. While they involve distinct molecular processes and give rise to different outcomes, the individual pathways are remarkably interconnected and may flexibly compensate for one another. In particular, diverse initiators and effectors involved in apoptosis or pyroptosis are interchangeably utilized to ensure cell death [31]. Although EMT cancer cells are generally resistant to apoptosis, they may still be susceptible to other cell death pathways, which may present therapeutic opportunities.

In the present study, we identified multiple genes encoding various AMPK subunits as transcriptional targets of the Snail transcription factors. Expression of AMPK genes was markedly repressed by EMT in multiple EMT cell models. Under energy stress conditions, EMT cancer cells failed to activate AMPK signaling and showed markedly increased cell death compared with non-EMT cells. Consistent with EMT-conferred resistance to apoptosis, EMT cancer cells instead mainly underwent pyroptosis under energy stress. The study suggests that EMT induces AMPK deficiency and renders cells vulnerable to energy stress.

## 2. Materials and Methods

### 2.1. Cell Culture

MCF7 (from ATCC) and previously established neuT and neuTemt cells [32] were cultured in Dulbecco’s Modified Eagle Medium (DMEM) supplemented with 10% fetal bovine serum (FBS) and penicillin-streptomycin. MCF10DCIS.com cells (obtained from Asterand) and DCIS-Snai1-ER cells [33] were maintained in DMEM/F12 supplemented with 5% horse serum. Glucose-, pyruvate- and glutamine-free DMEM was obtained from Gibco (catalog A14430). Glucose and glutamine were added to reconstitute low-glucose media. Metformin, phenformin, 2DG, BAY-876, disulfiram, ferrostatin, and necrostatin were purchased form Cayman Chemical.

### 2.2. RNA Extraction and Real-Time RT-PCR

Total RNA was extracted using TRIzol (Invitrogen) from cells in accordance with the manufacturer’s protocol. cDNA was generated using 1.5 μg of total RNA. Real-time PCR was carried out using SYBR green detection reagents (Applied Biosystems). Quantitative RT-PCR was conducted in triplicate; data represent the mean ± SD. The primers used in the study were:

AMPKα1: CGTGTACGAAGGAAGAATCC and (antisense) TGTGGAGTAGCAGTCCCTGA;

AMPKα2: ACCCACTGAAACGAGCAACT and (antisense) CAAGCTGGTCTTGAGGGTCA;

AMPKβ1: CCCAAAAGTGCTCCGATGTG and (antisense) CAGCGCGTATAGGTGGTTCA;

AMPKγ1: CCTTTAAACCGCTTGTCTGC and (antisense) GCCAATCTGTAGCTCTTCCA;

AMPKγ2: GATATCACGGTGACCCAGGC and (antisense) GCAGAATGTCCGACAGGGAA;

AMPKγ3: AAGATTTGGGCATCGGCACA and (antisense) CAAAGCGGGAATAGAGGCCC.

### 2.3. Western Blotting

Whole-cell lysates were resolved by SDS-PAGE gel electrophoresis, electrotransferred to a PVDF membrane, and probed with indicated antibodies. The following antibodies were purchased from Cell Signaling Technology: AMPKα1 (#2795), AMPKα2 (#2757), Phospho-AMPKα (Thr172) (#2535), AMPKβ1 (#4178), AMPKβ2 (#4148), AMPKγ1 (#4187), AMPKγ2, (#2536), AMPKγ3 (#2550), Acetyl-CoA Carboxylase (#3676), Phospho-Acetyl-CoA Carboxylase (Ser79) (#3661), cleaved Caspase-3 (Asp175) (#9661 and #9664), E-cadherin (#3195), Gasdermin D (#97558 and #93709), and cleaved Gasdermin D (Asp275) (#36425).

### 2.4. Chromatin Immunoprecipitation (ChIP)

Briefly, DCIS-Snai1-ER cells (±4HT for 2 days) were cross-linked with 1% formaldehyde for 10 min. The reaction was stopped by 0.125 M glycine solution. Cross-linked cells were washed in 1× phosphate-buffered saline (PBS) buffer and collected. Cell pellets were washed several times in washing buffer (0.25% TritonX-100, 10 mM EDTA, 0.5 mM EGTA, and 10 mM tris (pH 8.0)) and resuspended in sonication buffer (1 mM EDTA, 0.5 mM EGTA, and 10 mM tris (pH 8.0)), mixed with glass beads, and then subjected to the sonication process. The sonicated samples were diluted with ChIP buffer (0.01% SDS, 1.0% Triton X-100, 1.0 mM EDTA, 20 mM tris (pH 8.0), 150 mM NaCl) and incubated with IgG or antibodies against Snai1. The immunoprecipitates were subjected to a series of washing steps to remove nonspecific binding materials. After reverse cross-linking at 65 ℃ overnight, DNA was purified and then analyzed by real-time qPCR. The final results represent percentage of input chromatin, and error bars indicate S.D. from triplicate experiments. ChIP primers:

AMPKβ1: AATGGAATCGAGATAGCCTC and CTTTCCCAACCGCTTCAC;

AMPKγ2: ATGTAACTGCCCATCCTCG and CGAATTAAGAGTTCCATGCT;

AMPKγ3: GCATGGTGTCGAAGATGAC and GATGACGAACTGCGGAAAC.

### 2.5. Bioinformatic Analysis of ChIP-Seq Datasets

The assay was conducted as previously described [34]. ChIP-seq datasets were obtained from NCBI GEO (GSM1499414, GSE55421, GSE61475). Briefly, raw reads were trimmed with bbduk.sh (removal of adapters and low-quality reads), quality-evaluated by FastQC program, and aligned to human and mouse genomes (hg19 and mm9, respectively) using Bowtie2. Peaks were identified using the MACS2 program with parameters (high stringent cutoff q value <0.01) and annotated with the command “annotatePeaks.pl” from the HOMER package and GREAT. Genome browser tracks were created with the genomeCoverageBed command in BEDTools and normalized such that each value represents the read count per kilobase pair per million mapped and filtered reads, and data tracks of visualization were normalized to the number of fragments falling within all peaks for each sample. All sequencing tracks were visualized in the Integrative Genomics Viewer (IGV) genome browsers. De novo motif analysis was performed using “findmotifsgenome.pl” from the HOMER motif discovery algorithm.

### 2.6. Bioinformatic Analysis of RNA Expression Data

The indicated gene expression microarray data were downloaded and studied through clustering analysis of selected epithelial and mesenchymal markers that are positively or inversely co-expressed with AMPK genes in cancer cell lines and tumor samples. We next carried out a multi-gene score to normalize probeset log2 values with median values from all samples. These data were uploaded into Cluster 3.0 to hierarchically cluster the genes using “centered correlation” and “complete” as the distance metric and method. The output data were then loaded into Java Tree View software to generate a heatmap.

## 3. Results

### 3.1. Snail Binds to the Promoters of AMPK Genes

**Figure 1:**
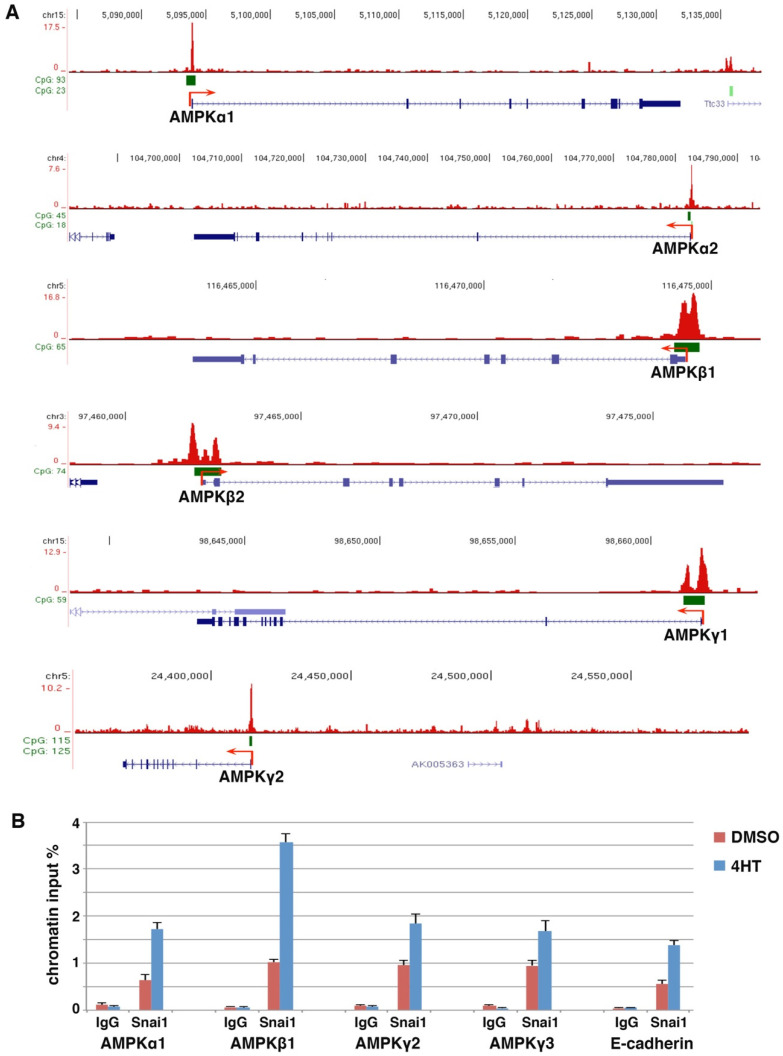
AMPK genes are direct transcriptional targets of Snai1. (**A**) Snai1 genomic binding in mouse MMTV-PyMT mammary carcinoma cells (ChIP-seq dataset GSM1499414) was visualized in the IGV genome browser. Exons (blue bars), CpG islands (green bars), and transcription start sites (red arrows). (**B**) ChIP analysis of Snai1 binding at selected AMPK gene promoters in DCIS-Snai1-ER cells (±4HT for 2 days). E-cadherin served as a positive control.

The Snail family transcription factors are considered master regulators of EMT and directly bind to the regulatory elements of a multitude of epithelial genes to repress their expression for EMT induction. We aimed to identify new genomic targets of Snail that may have important biological significance. Genome-wide binding of Snai1 and Snai2 in various cell types was previously investigated. The MMTV-PyMT transgenic mouse model develops mammary tumors with high incidence of Snai1-dependent spontaneous lung metastasis [6]. Therefore, we analyzed the published chromatin immunoprecipitation sequencing (ChIP-seq) data of Snai1 in the MMTV-PyMT mammary tumor cells [35]. We detected Snai1 binding at multiple genes encoding AMPK subunits. Most of these AMPK genes showed evident Snai1 binding around the transcription start sites (TSSs) (Figure 1A). Similarly, we explored available Snai2 ChIP-seq datasets in human epidermal progenitor cells [36] and differentiating embryonic stem cells [37]. Snai2 was also markedly enriched at a few AMPK gene promoters in these cells (Figure S1). These results suggest that mammalian AMPK genes are genomic targets of Snai1/2.

We performed standard ChIP assay to verify Snai1 binding at AMPK gene promoters in EMT cells. We previously stably expressed 4-hydroxytamoxifen (4HT)-inducible Snai1, a Snai1-estrogen receptor (Snai1-ER) fusion, in MCF10DCIS.com human basal breast epithelial cancer cells (referred to as DCIS) [33]. Upon 4HT binding, Snai1-ER translocates into the nucleus, binds to its genomic targets, and induces EMT. ChIP analysis confirmed that 4HT increased the binding of Snai1 at selected AMPK gene promoters in DCIS-Snai1-ER cells (Figure 1B). Collectively, the results suggest that AMPK genes are potentially direct Snail transcriptional targets.

### 3.2. Downregulation of AMPK Genes by EMT

**Figure 2:**
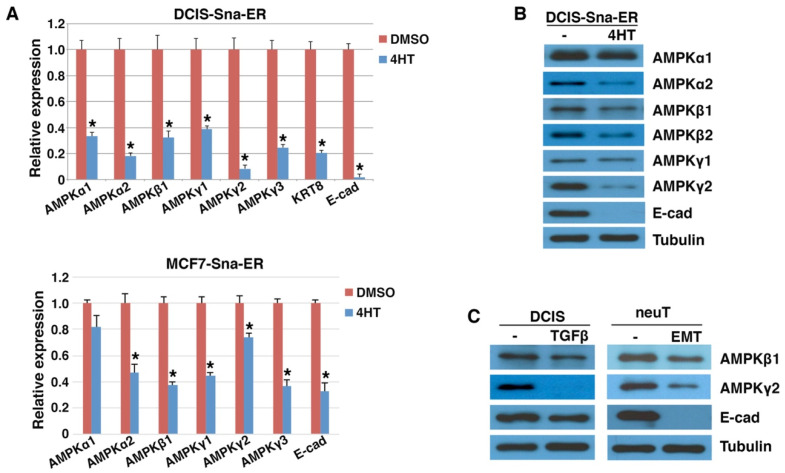
Downregulation of AMPK genes by EMT. (**A**) DCIS-Snai1-ER and MCF7-Snai1-ER human breast cancer cells were treated with vehicle (DMSO) or 4HT for 2 days, followed by quantitative RT-PCR analysis for AMPK genes (normalized by β-actin). Epithelial markers E-cadherin (E-cad) and keratin (KRT8) served as positive controls. Data shown as mean ± S.D. * *p* < 0.05. (**B**) Human DCIS-Snai1-ER cells were treated with DMSO or 4HT for 2 days, and subjected to immunoblotting with indicated antibodies. (**C**) DCIS breast epithelial cancer (±TGFβ for 2 days) and mouse neuT and neuTemt mammary tumor cells were immunoblotted with indicated antibodies. Tubulin was used as loading control.

AMPK is a central regulator of cellular metabolism, but the regulation of AMPK gene expression remains largely unexplored. Snai1/2 generally act as transcriptional repressors. Given their binding at the proximal promoters of AMPK genes, we investigated whether AMPK genes were repressed during EMT. Following Snai1 induction by 4HT, DCIS-Snai1-ER cells displayed morphological and gene expression changes characteristic of EMT [33]. Most AMPK genes were indeed markedly downregulated by 4HT in DCIS-Snai1-ER cells (Figure 2A). Similarly, when Snai1-ER was stably expressed in MCF7 human luminal breast cancer cells, AMPK genes were also repressed by 4HT (Figure 2A). Through immunoblotting, we found that multiple AMPK subunits (α1, α2, β1, β2, and γ2) were downregulated to various extents by 4HT in DCIS-Snai1-ER cells (Figure 2B). In particular, AMPKγ2 was strongly repressed along with E-cadherin by Snai1 (Figure 2B). AMPKγ1 was weakly expressed and AMPKγ3 was undetectable in these cells.

TGFβ induces the expression of virtually all EMT-TFs and is a potent EMT inducer in certain cultured carcinoma cells [1,38]. When DCIS cells were briefly exposed to TGFβ, before E-cadherin was fully repressed, AMPKγ2 expression was potently decreased and AMPKβ1 was also downregulated (Figure 2C). NeuT and neuTemt are a pair of isogenic mouse mammary carcinoma cell lines displaying epithelial and mesenchymal phenotypes, respectively [32]. Expression of AMPK β1 and γ2 was lowered in neuTemt cells compared with neuT cells (Figure 2C). Taken together, AMPK genes are repressed by EMT in multiple isogenic EMT cell models.

### 3.3. Expression of AMPK Genes in Human Cancer Cells Correlates with Epithelial Cell Markers

**Figure 3:**
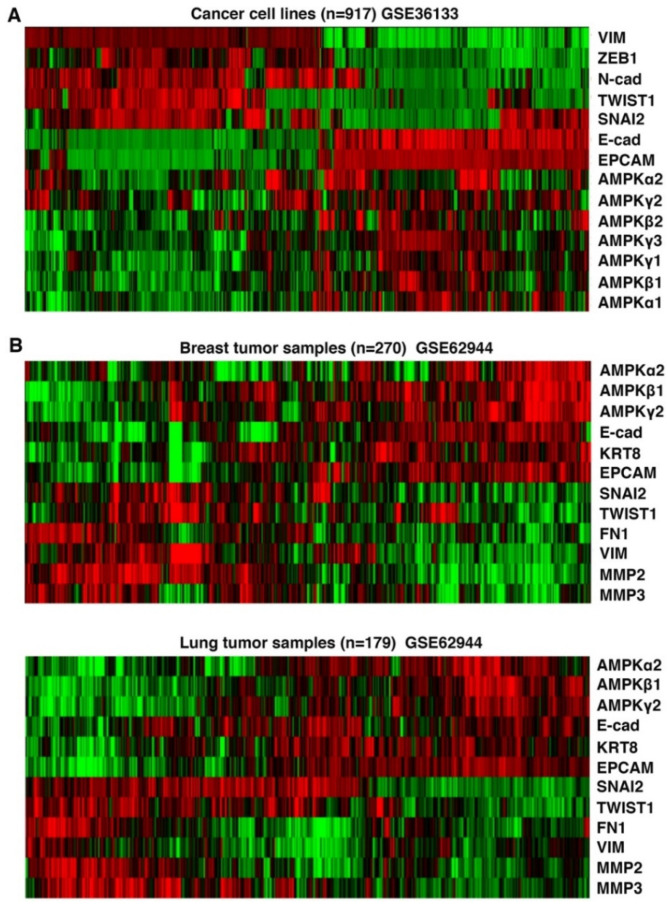
Correlation of AMPK gene expression with epithelial cell markers in human cancers. (**A**) Human cancer cell lines from the Cancer Cell Line Encyclopedia (CCLE) were clustered based on the expression levels of indicated AMPK genes, epithelial (E-cadherin and EPCAM) and mesenchymal (Vimentin, Zeb1, N-cadherin, Twist1, Snai2) markers. (**B**). Human breast and lung primary tumors were clustered based on expression levels of AMPK genes along with indicated epithelial markers and mesenchymal markers. Green and red colors indicate lower and higher expression, respectively.

**Figure 4:**
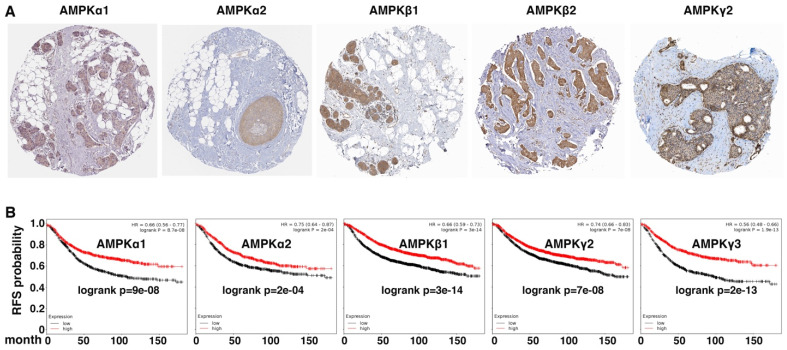
AMPK expression is enriched in epithelial cells in human cancers and is a prognostic indicator. (**A**) Immunohistochemistry analysis of indicated AMPK subunits in human breast tumor arrays. Data were from Human Protein Atlas (www.proteinatlas.org, accessed on 20 May 2022). (**B**) Kaplan–Meier survival was analyzed based on expression levels of AMPK genes in large cohorts of human breast cancer patients using online software (http://kmplot.com/analysis/, accessed on 20 May 2022). Log-rank *p* values are shown. Low and high expression of the indicated genes is represented by black and red, respectively. RFS: relapse-free survival.

We further evaluated the expression patterns of AMPK subunits in human cancer cell lines and primary tumor samples. Based on gene expression data in established human cancer cell lines from the Cancer Cell Line Encyclopedia (CCLE) portal (https://portals.broadinstitute.org/ccle/home, accessed on 20 May 2022), expression of AMPK genes correlated with epithelial cell markers (E-cadherin and EPCAM) and inversely with various mesenchymal cell markers (Figure 3A). In cohorts of human primary breast and lung tumor samples, expression of AMPK genes was heterogeneous, and was enriched in tumors exhibiting high expression of epithelial markers and low expression of mesenchymal markers (Figure 3B). Immunohistochemistry assays in human breast cancer samples showed that multiple AMPK subunits were enriched in epithelial cancer cells but absent in mesenchymal stroma (Figure 4A). Similar epithelial-enriched protein expression of AMPK subunits was observed in other human solid tumors, including lung, colon, and kidney cancers (www.proteinatlas.org). The epithelial-enriched expression of AMPK genes is consistent with their repression by EMT.

As the EMT gene expression signature is associated with adverse prognosis, we examined whether AMPK gene expression in human cancers was prognostic. In a large cohort of breast cancer patients [39], high expression of AMPK genes robustly correlated with increased relapse-free survival (Figure 4B). Similarly, in a human lung cancer cohort [39], elevated expression of multiple AMPK genes was associated with favorable prognosis (data not shown). Overall, high expression of AMPK genes in human cancer indicates favorable clinical outcomes.

### 3.4. EMT Impairs Energy-Stress-Induced AMPK Activation

Given the downregulation of multiple AMPK genes during EMT, especially the γ regulatory subunit that directly senses AMP and ADP levels, we determined whether energy-stress-induced activation of AMPK might be impaired by EMT. A well-established indicator of AMPK activation is the phosphorylation of the conserved threonine 172 (T172) in the α subunit kinase domain, primarily mediated by LKB1 [26]. Activated AMPK, in turn, inhibits fatty acid synthesis and promotes fatty acid oxidation by phosphorylating serine 79 (S79) of acetyl-CoA carboxylase (ACC), the rate-limiting enzyme in fatty acid synthesis and oxidation [26].

**Figure 5:**
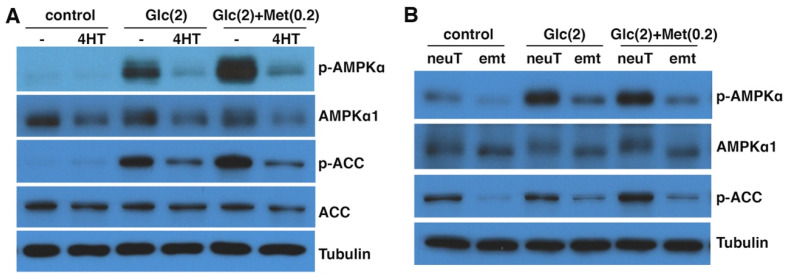
EMT suppresses AMPK activation by energy stress. (**A**) DCIS-Snai1-ER cells were treated with vehicle or 4HT for 2 days, then placed in normal (control) or low-glucose (Glc) media ± metformin (Met) for 6 h, followed by immunoblotting for phospho- or total AMPKα and ACC. (**B**) NeuT and neuTemt cells were under normal (control) or low glucose (Glc) media ± metformin (Met) for 6 h, then subjected to immunoblotting. Numbers in parentheses indicate concentrations (in mM). *p*-AMPKα: T172-phospho AMPKα. *p*-ACC: S79-phospho ACC. Glutamine levels in low-glucose media in (**A**) and (**B**) were 2 and 0 mM, respectively.

In DCIS-Snai1-ER cells in normal culture media, the basal levels of AMPK activation were essentially undetectable. When the cells were exposed to the low-glucose condition for 6 h, phosphorylation of AMPKα T172 and ACC S79 was markedly induced (Figure 5A), indicating activation of AMPK signaling. For DCIS-Snai1-ER cells with 4HT-induced EMT, basal activation of AMPK in normal media was also negligible; however, low-glucose-induced AMPK activation was mostly dampened (Figure 5A). The diabetes therapeutic biguanides metformin and its more potent analog, phenformin, inhibit mitochondrial respiratory chain complex I and ATP synthesis, thus resulting in energy stress and indirectly activating AMPK [40,41,42]. The effect of metformin is enhanced by low glucose levels [43]. When metformin was combined with low glucose, DCIS-Snai1-ER cells displayed even stronger phosphorylation of AMPKα and ACC, yet the 4HT-induced EMT cells remained largely irresponsive regarding AMPK activation (Figure 5A).

Similarly, we compared AMPK activation in neuT vs. neuTemt cells under energy stress. In normal media, neuT cells exhibited higher basal levels of AMPK activation than neuTemt cells (Figure 5B). In low-glucose media without or with metformin, AMPK was activated in neuT cells but not in neuTemt cells (Figure 5B). We further tried a variety of metabolic stress conditions, including changes in glucose and glutamine levels and the use of phenformin. These energy stress settings generally increased phosphorylation of AMPKα and ACC in neuT cells, but such stress-induced phosphorylation events were blunted in neuTemt cells (Figure S2). Taken together, the results suggest that energy-stress-mediated activation of AMPK signaling is blocked by EMT.

### 3.5. EMT Sensitizes Cells to Energy Stress

**Figure 6:**
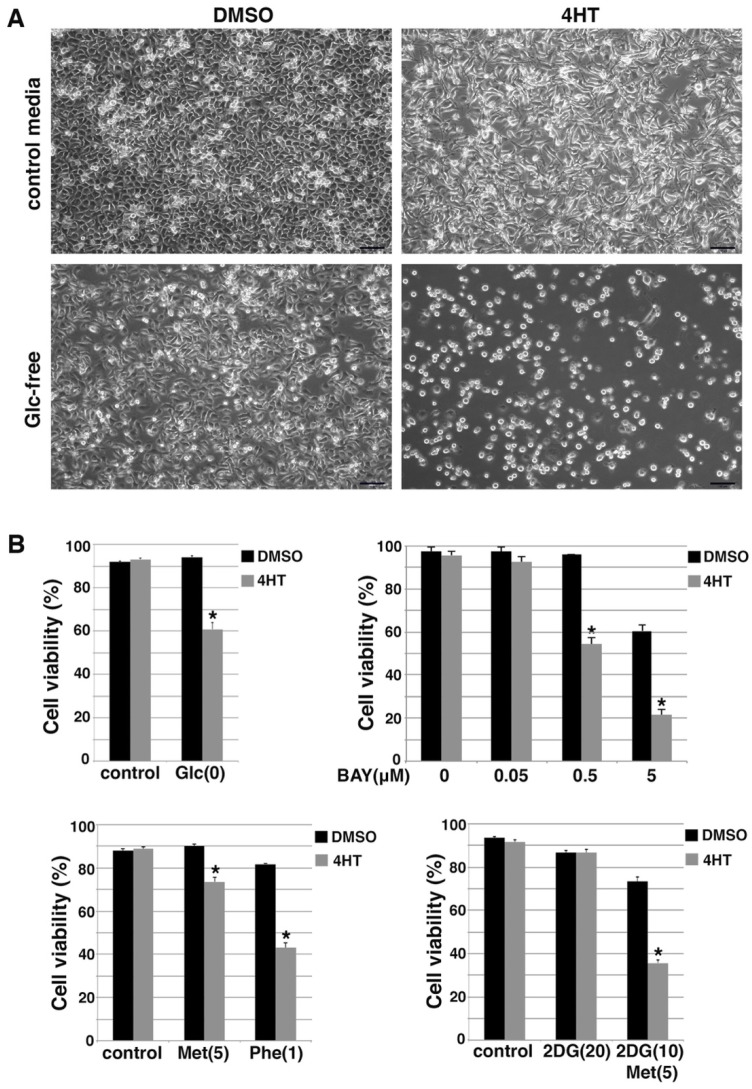
Snail-driven EMT sensitizes DCIS-Snai1-ER cells to energy stress conditions. (**A**) DCIS-Snai1-ER cells were treated with DMSO or 4HT for 2 days, then cultured in normal or glucose (Glc)-free media for 1 day. (**B**) Trypan blue cell viability assays. DCIS-Snai1-ER cells were treated with DMSO (vehicle) or 4HT for 2 days, followed by 1-day treatment with glucose (Glc) deprivation, glucose transporter inhibitor BAY-876 (Bay), metformin (Met, 5mM) or phenformin (Phe, 1 mM), or 2DG (10, 20 mM) ± metformin (5 mM). Cell viability was calculated as the number of viable cells divided by the total number of cells. Data shown as mean ± S.D. * *p* < 0.01.

Because EMT suppressed energy-stress-induced AMPK activation that is key to cell survival, we determined whether EMT cancer cells were more vulnerable to energy stress conditions than their isogenic non-EMT counterparts. Glucose starvation causes energy deficiency. When DCIS-Snai1-ER cells were cultured in glucose-free media overnight, the total cell number slightly decreased and there were very few dead cells (Figure 6A). In striking contrast, under the same glucose deprivation condition, 4HT-induced EMT cells showed dramatically reduced cell number, and most cells rounded up and started to detach or were afloat, indicating profound ongoing cell death (Figure 6A). Trypan blue exclusion assay validated that under glucose starvation, ~40% of the EMT cells were dead, whereas non-EMT cells showed minimal cell death (Figure 6B).

We further introduced energy stress with pharmacological compounds. Glucose transporter type 1 (GLUT1) is a key rate-limiting factor in the transport of glucose in cancer cells [44]. BAY-876 is a potent and selective GLUT1 inhibitor [45]. BAY-876 was able to decrease cellular ATP levels and activate AMPK [46]. We used BAY-876 to inhibit glucose uptake in the DCIS-Snai1-ER cells. When treated with increasing concentrations of BAY-876, 4HT-induced EMT cells exhibited much more cell death than non-EMT DCIS-Snai1-ER cells (Figure 6B). For instance, BAY-876 at 0.5 μM had no effect on the viability of control non-EMT cells, but killed nearly 50% of the EMT cells (Figure 6B). Moreover, we treated DCIS-Snai1-ER cells with metformin and phenformin. Both compounds caused more cell death in EMT cells than non-EMT cells, and phenformin was more potent than metformin in regard to killing EMT cells (Figure 6B). Glucose analog 2-deoxy-glucose (2DG) inhibits glycolysis [47] and enhances AMPK activation by metformin [48,49]. 2DG alone had little influence on the survival of EMT and non-EMT DCIS-Snai1-ER cells, but a combination of 2DG and metformin potently induced cell death in EMT cells while exerting only a mild effect on non-EMT cells (Figure 6B).

We validated the results with a different dye exclusion assay. Propidium iodide (PI) is a membrane impermeant red-fluorescent nuclear dye that is excluded from viable cells with intact membranes and thus selectively stains only dead cells. Hoechst 33342 (HO) is a blue-fluorescent dye that can readily cross cell membranes to stain both living and dead cells. Dual staining with PI/HO unambiguously reveals percent cell death in a cell population. EMT and non-EMT DCIS-Snai1-ER cells in normal media showed little cell death (Figure S3). When placed in low-glucose and glutamine-free media, EMT cells showed markedly more cell death than non-EMT cells (Figure S3). Further addition of metformin induced even more cell death, preferentially in EMT cells (Figure S3). Taken together, the results suggest that Snail-induced EMT renders cells hypersensitive to energy stress.

**Figure 7:**
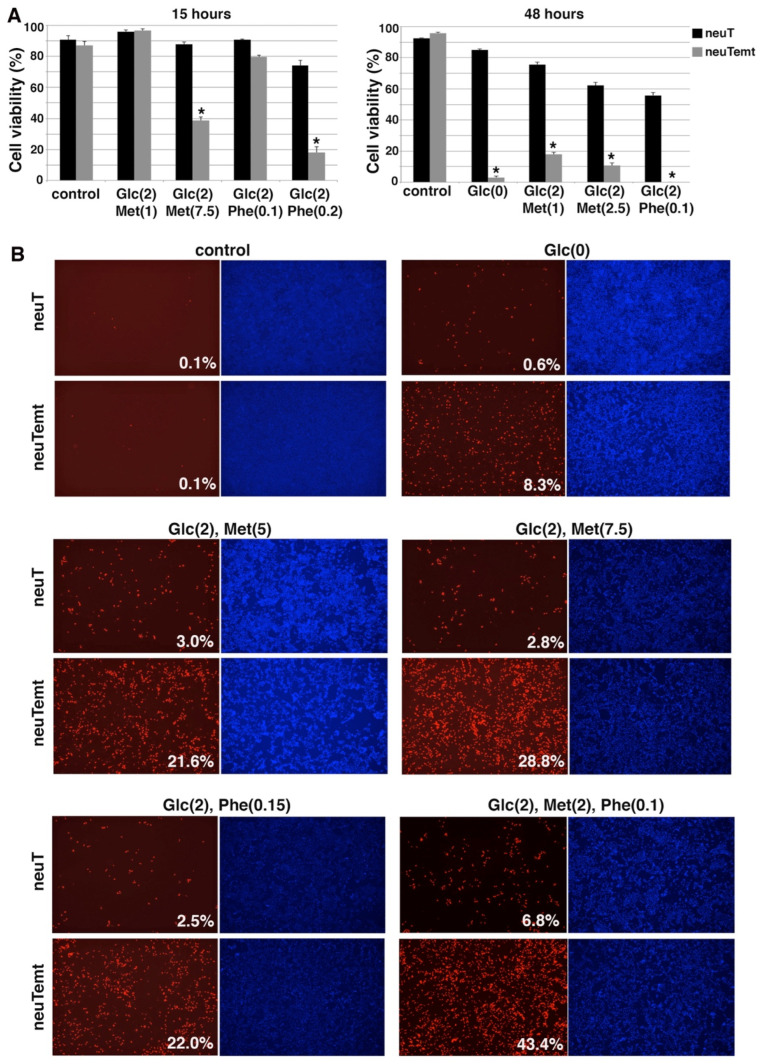
EMT cells are hypersensitive to various energy stress conditions. (**A**). NeuT and neuTemt cells were subjected to glucose deprivation or low glucose ± metformin or phenformin for 15 and 48 h. Cell viability was determined by trypan blue exclusion assay. (**B**). NeuT and neuTemt cells were subjected to indicated metabolic stress for 15 h, and stained with PI/HO. Percent dead cells (red/blue) was quantified with ImageJ. Numbers in parentheses are concentrations (in mM). Glc: glucose. Met: metformin. Phe: phenformin. Data shown as mean ± S.D. * *p* < 0.01.

We next verified whether TGFβ-induced EMT also sensitized cells to energy stress. Under various stress conditions (generated by glucose deprivation or the use of 2DG and metformin), TGFβ-treated DCIS cells showed markedly more cell death than control non-EMT cells (Figure S4). The hypersensitivity to energy stress was also confirmed in the EMT model of neuT cells. NeuT tumors were driven by the transgenic oncogene neu (rodent homolog of HER2). NeuT cells were sensitive to lapatinib, a small-molecule dual inhibitor targeting HER2 and EGFR [50] (Figure S5), suggesting that neuT cells are still dependent on the original oncogenic driver. By contrast, neuTemt cells were resistant to lapatinib (Figure S5), which is consistent with the notion that EMT confers resistance to targeted therapies. However, under various energy stress conditions, including glucose deprivation or low glucose in combination with varying concentrations of metformin or phenformin, drug-resistant neuTemt cells always exhibited markedly more cell death than neuT cells (Figure 7A,B). Collectively, the results from multiple EMT cell models suggest that EMT cancer cells are substantially more vulnerable to energy stress than their isogenic non-EMT counterparts.

### 3.6. EMT Induces Pyroptotic Cell Death under Energy Stress

**Figure 8:**
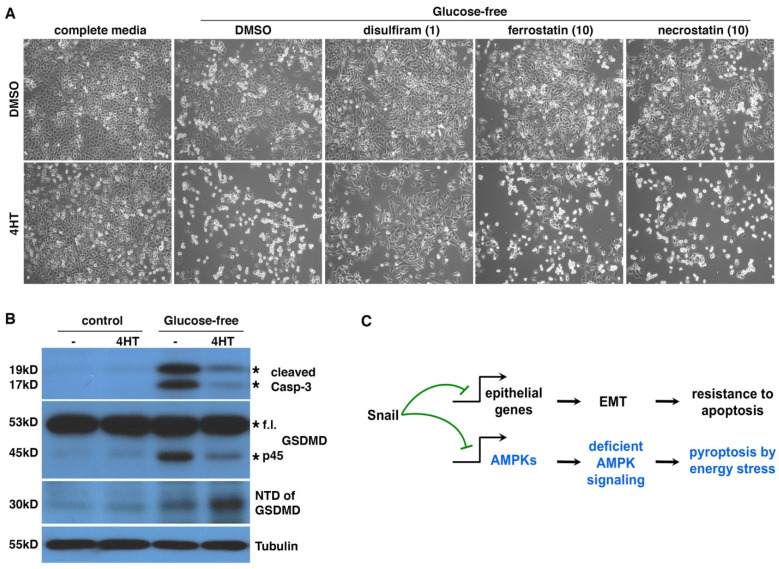
Energy stress induces pyroptosis in EMT cancer cells. (**A**) Disulfiram blocked glucose starvation-caused cell death in EMT cells. DCIS-Snai1-ER cells were treated with 4HT or vehicle (DMSO) for 2 days, followed by glucose starvation [Glc(0)] ± disulfiram (1μM), ferrostatin-1 (10 μM), or necrostatin-1 (10 μM) for 1 day. The three compounds inhibit pyroptosis, ferroptosis, and necroptosis, respectively. Lower concentrations of ferrostatin-1 and necrostatin-1 also failed to rescue cells (data not shown). (**B**) GSDMD cleavage in EMT and non-EMT cells under glucose starvation. DCIS-Snai1-ER cells were treated with 4HT or vehicle (DMSO) for 2 days, followed by glucose starvation for 1 day, then subjected to immunoblotting with indicated antibodies. Expected protein bands are denoted by asterisks. F.l. full length. (**C**) EMT switches energy stress-induced cell death forms. As Snail independently represses diverse genomic targets, EMT inhibits apoptosis and promotes pyroptosis in cells under energy stress by suppressing AMPK signaling.

The nature of EMT cell death under energy stress was unclear. Despite pronounced cell death under glucose starvation (Figure 6A), DCIS-Snai1-ER EMT cells did not show increases in caspase-3/7 activity (data not shown). This is consistent with EMT-driven resistance to apoptosis. There are multiple forms of programmed necrosis, including pyroptosis, necroptosis, and ferroptosis [31]. We probed the mechanism underlying EMT cell death under energy stress using chemical inhibitors of each cell death program. We found that disulfiram, a potent inhibitor of pyroptosis by blocking gasdermin D (GSDMD) pore formation [51], completely suppressed the death of DCIS-Snai1-ER EMT cells under glucose starvation, whereas ferrostatin (a ferroptosis inhibitor) and necrostatin (a necroptosis inhibitor) had a mild and no effect on cell viability, respectively (Figure 8A). The cells rescued by disulfiram retained a mesenchymal morphology (Figure 8A), suggesting that disulfiram blocks EMT cell death not by reversing EMT. Disulfiram also appeared to have no effect on the growth arrest caused by energy stress. The results suggest that pyroptosis is potentially the primary pathway mediating EMT cell death under energy stress.

Pyroptosis is usually caused by the cleavage of GSDMD by inflammatory caspases (e.g., caspase-1/4/5) to generate a 30KD N-terminal domain (NTD), which oligomerizes and forms large pores in the plasma membrane, eventually leading to cell death [52]. Such pore formation allows cellular uptake of PI, which is consistent with extensive PI staining in EMT cells under energy stress (Figure S3 and Figure 7B). We examined apoptosis markers and GSDMD cleavage in DCIS-Snai1-ER cells with or without EMT under energy stress. Caspase-3 cleavage is a hallmark of apoptosis. One day under glucose starvation, non-EMT cells mostly survived (Figure 6A and Figure 8A) but started showing cleaved caspase-3 (Figure 8B), indicating the initiation of apoptosis. By contrast, although EMT cells under glucose deprivation showed strong cell death (Figure 6A and Figure 8A), caspase-3 cleavage was barely detectable (Figure 8B), confirming that the cells do not die from apoptosis. For GSDMD in cells under energy stress, non-EMT cells generated a 45KD fragment, but not the pyroptotic 30KD NTD (Figure 8B). It was reported that active apoptotic caspase-3/7 block pyroptosis by cleaving GSDMD at a distinct site from the inflammatory caspases to produce a 45KD inactive fragment [53]. It is conceivable that the 45KD fragment in non-EMT cells resulted from GSDMD cleavage by apoptotic caspases. In contrast, in EMT cells, energy stress increased the yield of GSDMD NTD (Figure 8B), indicative of pyroptosis. Taken together, under energy stress, non-EMT cells activate caspase-3/7 to initiate apoptosis and actively suppress pyroptosis by cleaving GSDMD into an inactive form, whereas EMT cells do not activate apoptotic caspases but instead proteolytically activate GSDMD to induce pyroptosis. Therefore, EMT switches energy-stress-induced cell death forms from apoptosis to pyroptosis (Figure 8C).

## 4. Discussion

### 4.1. Identifying and Targeting EMT-Induced Therapeutic Vulnerabilities

EMT is a developmental program that is hijacked by cancer cells to exacerbate malignancy. The key to targeting EMT cancer cells is to uncover potential EMT-induced specific vulnerabilities. Snail family transcription factors are master EMT drivers and directly repress the transcription of a great number of downstream target genes. Epithelial genes constitute only a subset of Snail transcription targets. There exist plenty of other types of genomic targets that convey important biological activities. For instance, Snai2 directly represses puma [54], a critical BH3-only pro-apoptotic factor. This regulation conceivably may contribute to EMT-associated resistance to apoptosis. In the present study, multiple genes encoding AMPK complex subunits were identified as Snail genomic targets. Accordingly, AMPK genes were downregulated by EMT, and activation of AMPK signaling in response to energy stress was impaired in EMT cancer cells. Given the prevalent role of Snail transcription factors in EMT, deficient AMPK activation is probably a common byproduct and characteristic of EMT. It was recently reported that downregulation of AMPKα1 expression in breast cancer was associated with poor prognosis [55]. Repression of AMPK genes by Snail is thus consistent with EMT-mediated tumor progression. However, the role of AMPK in cancer is context-dependent and AMPK may promote or suppress cancer [56,57]. AMPK signaling is vital for cancer cells to reprogram cellular metabolism to survive energy stress conditions. Cancer cells deficient in AMPK signaling are unable to properly respond to and survive energy stress [28,29,30]. Therefore, by suppressing AMPK, EMT renders cancer cells vulnerable specifically to energy stress. This finding suggests that although EMT promotes malignant progression, it also concomitantly induces vulnerabilities that may be therapeutically exploited to selectively eradicate EMT cancer cells.

Pharmacological compounds that inhibit glucose uptake or mitochondrial bioenergetics generate energy stress. GLUT1 inhibitor BAY-876 was quite effective against EMT cancer cells in vitro (Figure 6B). Metformin and phenformin inhibit mitochondrial ATP synthesis [41,42]. Metformin, the most widely prescribed drug for type 2 diabetes, has demonstrated anticancer activities [58]. Metformin requires transporters for cell entry, which may restrict its primary targets to specific cell types [59]. In our in vitro assays, the potency of metformin alone against EMT cancer cells was weak, but was enhanced by low-glucose conditions [43]. As most tumors have a low-glucose microenvironment, metformin could be potent in inducing energy stress in vivo. Given its proven safety and a wealth of experience on its use, repurposing metformin to target EMT cancer cells is an attractive therapeutic avenue. However, recent clinical trials failed to support the administration of adjuvant metformin in breast cancer [60,61,62,63]. By contrast, a meta-analysis found that metformin adjunct with standard cancer therapies significantly improved lung cancer patient survival [64]. It remains unclear if the difference may be attributed to differential metformin transporter expression in the two cancer types. Of note also is that these clinical studies were not designed to specifically target EMT cancer cells. Phenformin readily permeates cells and shows much greater potency than metformin. Phenformin was withdrawn for clinical use for diabetes due to incidence of lactic acidosis. This side effect may be acceptable for using phenformin against EMT cancer cells due to the short duration of treatment. New OXPHOS inhibitors such as IACS-010759 and Gboxin [65,66] may also be useful in targeting EMT cancer cells. We have tested a variety of energy stress conditions in vitro, and animal studies are needed to identify effective energy stress inducers and determine their efficacy in targeting EMT cancer cells in vivo.

It should be emphasized that it is energy stress, but not AMPK activation, that preferentially kills EMT cells over non-EMT cells. Direct AMPK agonists that do not generate energy stress are unlikely to kill EMT cells but instead may protect them. In addition, EMT is usually partial, producing cells with hybrid epithelial–mesenchymal traits. Such partial EMT cells may still retain sufficient residual AMPK signaling activities (albeit decreased compared with non-EMT cells) and are not hypersensitive to energy stress. Pharmacological AMPK inhibitors may be used to further shut down AMPK activity in these cells and effectively sensitize them to energy stress inducers.

### 4.2. EMT Switches Energy-Stress-Induced Cell Death Forms

Apoptosis is a highly regulated, energy-demanding process involving a number of ATP-dependent steps, such as caspase activation, enzymatic hydrolysis of macromolecules, chromatin condensation, bleb formation, and apoptotic body formation [67]. Apoptosis requires intracellular ATP for the execution of the cell death program, and depletion of ATP causes switching of the form of cell death, from apoptosis to necrosis [68,69]. In response to energy stress, epithelial cells are able to adaptively activate AMPK signaling to stimulate catabolism and autophagy to restore bioenergetic homeostasis and maintain survival. If energy stress is prolonged and too severe to resolve, the AMPK metabolic program presumably allows the cells to generate enough ATP to enact apoptotic cell death [70]. Activated apoptotic caspases also inactivate GSDMD by cleavage to prevent pyroptosis [53]. In contrast, EMT is well-known to confer increased resistance to apoptosis. Furthermore, due to deficient AMPK activation under energy stress, EMT cells may fail to produce enough ATP to execute the apoptosis program and, instead, undergo necrotic cell death. Therefore, under energy stress, epithelial cells tend to undergo apoptosis, and EMT cells prefer necrosis.

It remains to be elucidated how pyroptosis is induced in EMT cells under energy stress. Inflammasomes activate inflammatory caspases and subsequently pyroptosis. The NLRP3 inflammasome senses a broad range of stimuli. Reactive oxygen species (ROS) serve as important signals for NLRP3 inflammasome activation, although the precise underlying mechanism still needs to be addressed [71,72,73,74]. The efficacy of GSDMD cleavage is reduced by attenuation of ROS production. After GSDMD cleavage, ROS promote NTD oligomerization and pore formation [75]. Overall, ROS stimulate pyroptosis. Energy stress conditions also increase oxidative stress. AMPK signaling critically enhances antioxidant defense to mitigate oxidative stress and restore redox balance [29,76]. Therefore, under energy stress, EMT cells may accumulate excessive ROS that activate inflammasomes, leading to pyroptotic cleavage of GSDMD.

It was recently reported that AMPK deficiency sensitizes cells to ferroptosis [77], an iron-dependent form of regulated necrosis that is induced by the overproduction of phospholipid hydroperoxides [78]. AMPK-deficient cells alter fatty acid metabolism and may aberrantly accumulate fatty acids, including polyunsaturated fatty acids (PUFAs), which facilitate ferroptosis. On the other hand, high mesenchymal state cells (including some therapy-resistant cancer cells) and certain EMT cells (in particular, those induced by Zeb1) are hypersensitive to ferroptosis inducers [79,80]. One may thus expect that EMT cells under energy stress may die from ferroptosis. However, in our assay, a ferroptosis inhibitor failed to strongly suppress DCIS-Snai1-ER EMT cell death under glucose starvation (Figure 8A). Moreover, EMT did not affect the sensitivity of DCIS-Snai1-ER cells to ferroptosis inducers (e.g., erastin, ML-210) (data not shown). It is worth noting that in these reports, ferroptosis induction was not under energy stress conditions. As ROS appear to be a common factor implicated in activation of both pyroptosis and ferroptosis, it is possible that EMT may sensitize cells to both necrotic forms, and the exact death program depends on the death-inducing signals. When exposed to ferroptosis-inducing compounds, certain EMT cells preferentially undergo ferroptosis; when under energy stress, EMT cells mainly undertake pyroptosis.

Different forms of cell death elicit distinct host immune responses [81,82]. Pyroptotic cells release intracellular damage-associated molecular patterns (DAMPs) (e.g., HMGB1), the messengers of immunogenicity [81,82]. Therefore, unlike apoptosis, pyroptosis is immunologically stimulatory and hyperinflammatory, and may act as an enhancer for immunotherapy [83]. EMT leads to a tumor immunosuppressive microenvironment and resistance to immunotherapy [84,85]. It is interesting to verify if energy stress that causes pyroptosis in EMT cancer cells may enhance immunotherapy efficacy.

## 5. Conclusions

The Snail EMT-driving transcription factors repress AMPK gene expression, resulting in AMPK deficiency in EMT cancer cells. As AMPK signaling is critical for cell survival under energy stress, EMT cancer cells become hypersensitive to energy stress conditions and undergo pyroptosis. Therefore, although EMT confers enhanced malignant properties including increased resistance to apoptosis, the process concomitantly renders cancer cells susceptible to pyroptosis. The EMT-induced vulnerability may allow specific targeting of EMT cancer cells.

## 6. Limitations of the Study

EMT was reported to be highly variable and vastly context-specific, and no common EMT-defining gene expression program could be identified [86]. Although AMPK downregulation was observed in a few EMT models in this study, it awaits future studies to determine whether this regulation is pervasive in EMT. In addition, the current work was limited to cell culture models in vitro. It remains to be investigated if AMPK signaling is substantially impaired by EMT and if energy stress, introduced by pharmacological compounds that target bioenergetic pathways or non-pharmacological approaches (e.g., dietary interventions, intermittent fasting, exercise), effectively eradicates EMT cancer cells in vivo.

## Supplementary Materials

The following supporting information can be downloaded at: https://www.mdpi.com/article/10.3390/cells11142208/s1, Figure S1: Genomic binding of Snai2 at indicated loci in human epidermal progenitor cells (skin, GSE55421) and differentiating embryonic stem cells (ES, GSE61475). Figure S2. EMT impairs activation of AMPK signaling by energy stress. (**A**). NeuT and neuTemt cells were under normal (control) or low glucose (Glc) media ± metformin (Met) for 6 h, then subjected to immunoblotting with indicated antibodies. (**B**). NeuT and neuTemt cells were under normal (con) or indicated stress conditions for 6 h and subjected to immunoblotting for indicated proteins. Glc: glucose; Gln: glutamine; M: metformin; P: phenformin. Numbers in parentheses indicate concentrations (for Glc, Gln and M: mM; for P: μM). Glucose concentrations in (B) 4 mM. Figure S3. Snail-driven EMT cells are hypersensitive to metabolic stress. DCIS-Snai1-ER cells were treated with DMSO or 4HT for 2 days, then subjected to indicated metabolic stress conditions for 14 h, and stained with PI/HO. Percentage of dead cells was quantified with ImageJ (red/blue) and is shown. Control: normal media. Glc: glucose. Gln: Glutamine. Met: metformin. Numbers in parentheses indicate concentrations (in mM). Figure S4. TGFβ-induced EMT renders cells hypersensitive to energy stress. DCIS cells were exposed to TGFβ for 2 days, followed by glucose starvation [Glc(0)] or metformin ± 2DG treatment for 1 day. Numbers in parentheses indicate concentrations (in mM). Cell viability was determined by trypan blue. Data shown as mean ± S.D. * *p* < 0.01. Figure S5. NeuTemt cells resist lapatinib. NeuT and neuTemt cells were treated with increasing concentrations of lapatinib for 4 days (left) or lapatinib (5 μM) for up to 3 days (right). Cell viability refers to percent living cells in the population; relative growth indicates the increases of living cell numbers.
