# Supplementary material for: Epithelial–Mesenchymal Transition Suppresses AMPK and Sensitizes Cancer Cells to Pyroptosis under Energy Stress
Source: Cells. 2022 Jul 15;11(14):2208. doi: 10.3390/cells11142208 (PMC9322750; doi:10.3390/cells11142208)
Supplement: Supplementary file 1 [file cells-11-02208-s001.zip › cells-1761839-supplementary.pdf]

Supporting data

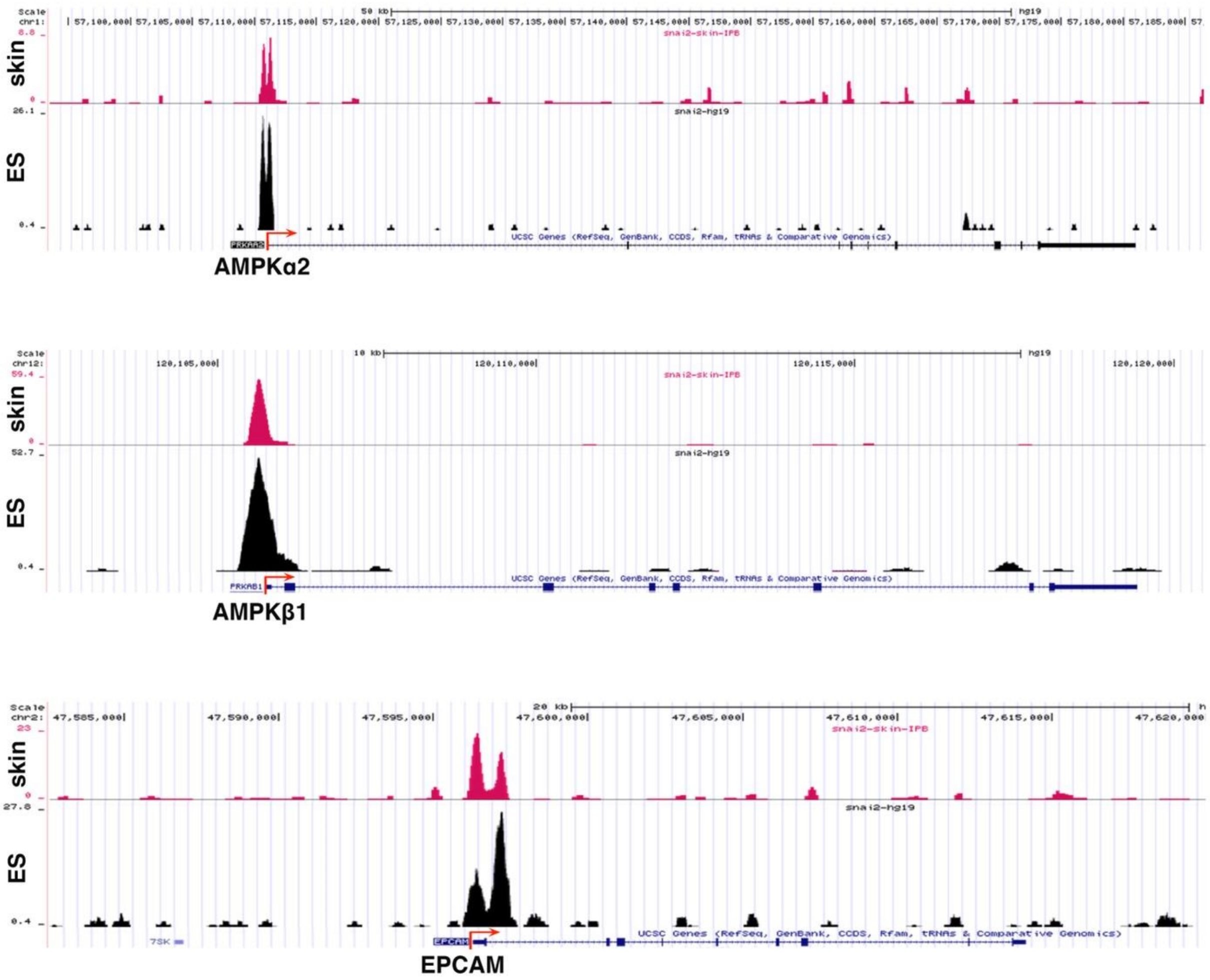

Figure S1. Genomic binding of Snai2 at indicated loci in human epidermal progenitor cells (skin, GSE55421) and differentiating embryonic stem cells (ES, GSE61475).

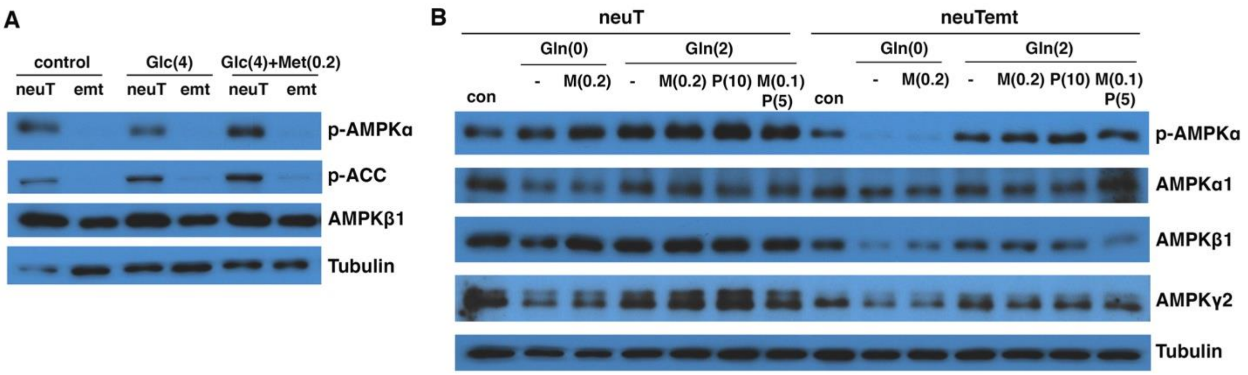

Figure S2. EMT impairs activation of AMPK signaling by energy stress.
(A). NeuT and neuTemt cells were under normal (control) or low glucose (Glc) media ± metformin (Met) for 6 hours, then subjected to immunoblotting with indicated antibodies. (**B**). NeuT and neuTemt cells were under normal (con) or indicated stress conditions for 6 hours and subjected to immunoblotting for indicated proteins. Glc: glucose; Gln: glutamine; M: metformin; P: phenformin. Numbers in parentheses indicate concentrations (for Glc, Gln and M: mM; for P: μM). Glucose concentrations in (**B**) 4 mM.

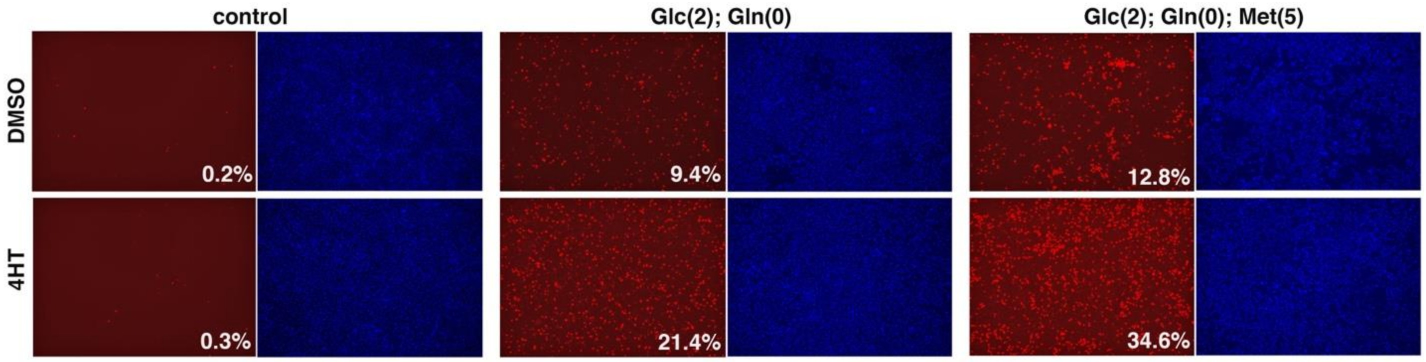

Figure S3. Snail-driven EMT cells are hypersensitive to metabolic stress. DCIS-Snai1-ER cells were treated with DMSO or 4HT for 2 days, then subjected to indicated metabolic stress conditions for 14 hours, and stained with PI/HO. Percentage of dead cells was quantified with ImageJ (red/blue) and is shown. Control: normal media. Glc: glucose. Gln: Glutamine. Met: metformin. Numbers in parentheses indicate concentrations (in mM).

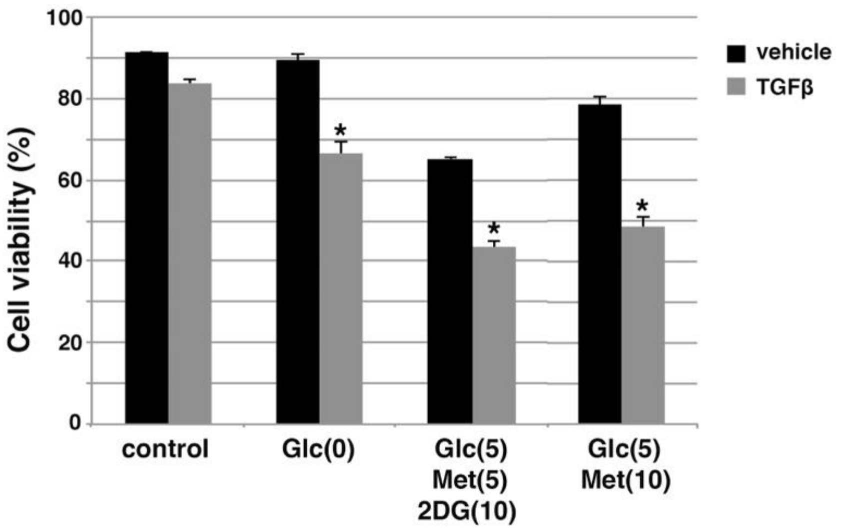

Figure S4. TGFβ-induced EMT renders cells hypersensitive to energy stress. DCIS cells were exposed to TGFβ for 2 days, followed by glucose starvation [Glc(0)] or metformin ± 2DG treatment for 1 day. Numbers in parentheses indicate concentrations (in mM). Cell viability was determined by trypan blue. Data shown as mean ± S.D. * $p < 0.01$.

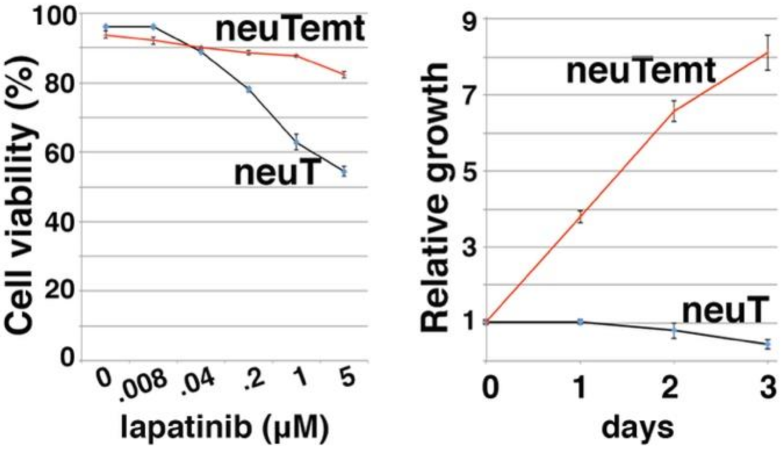

Figure S5. NeuTemt cells resist lapatinib. NeuT and neuTemt cells were treated with increasing concentrations of lapatinib for 4 days (left) or lapatinib (5μM) for up to 3 days (right). Cell viability refers to percent living cells in the population; relative growth indicates the increases of living cell numbers.
